# Highly efficient patterning technique for silver nanowire electrodes by electrospray deposition and its application to self-powered triboelectric tactile sensor

**DOI:** 10.1038/s41598-021-01043-6

**Published:** 2021-11-02

**Authors:** Jin Yeong Song, Jae Hee Oh, Dongwhi Choi, Sang Min Park

**Affiliations:** 1 School of Mechanical Engineering, Pusan National University, 63-2 Busan University-Ro, Geumjeong-gu, Busan, 46241 South Korea; 2 Department of Mechanical Engineering (Integrated Engineering Program), Kyung Hee University, 1732 Deogyeong-daero, Yongin, Gyeonggi 17104 South Korea

**Keywords:** Nanoscale materials, Nanowires

## Abstract

A patterned transparent electrode is a crucial component of state-of-the-art wearable devices and optoelectronic devices. However, most of the patterning methods using silver nanowires (AgNWs), which is one of the outstanding candidate materials for the transparent electrode, wasted a large amount of unused AgNWs during the patterning process. Here, we report a highly efficient patterning of AgNWs using electrospray deposition with grounded electrolyte solution (EDGE). During electrospray deposition, a patterned electrolyte solution collector attracted AgNWs by strong electrostatic attraction and selectively deposited them only on the patterned collector, minimizing AgNW deposited elsewhere. The enhanced patterning efficiency was verified through a comparison between the EDGE and conventional process by numerical simulation and experimental validation. As a result, despite the same electrospray deposition conditions for both cases except for the existence of the electrolyte solution collector, the coverage ratio of AgNWs fabricated by the EDGE process was at least six times higher than that of AgNWs produced by the conventional process. Furthermore, the EDGE process provided high design flexibility in terms of not only the material of the substrate, including a polymer and a ceramic but also the shape of the substrate, including a 2D flat and 3D curved surface. As an application of the EDGE process, a self-powered touch sensor exploiting the triboelectric effect was demonstrated. Thus, the EDGE process would be utilized in further application in wearable or implantable devices in the field of biomedicine, intelligent robots, and human–machine interface.

## Introduction

Over the past decade, the advance of transparent electrode technology enabled to development of novel optoelectronic devices and energy harvesting devices such as touch screen sensors, capacitive strain sensors, organic light-emitting diodes (OLEDs), transparent heaters, and solar cells^1–5^. Indium tin oxide (ITO) has long been used in both research and industrial fields for optoelectronic and energy harvesting applications. Despite the superior electrical conductivity and visual transparency of ITO, the intrinsic brittleness of ITO has been motivated the research group to discover alternative material against ITO’s vulnerability. Representing substitutions for ITO are carbon nanotubes(CNTs)^6,7^, graphene^3,8^, metal nanofibers^9,10^, and conducting polymers^11,12^. Among other candidates, silver nanowire (AgNW) is a prominent alternative material in light of its sustainability under various and repeated deforming conditions. Furthermore, its superior optical transparency and electrical conductivity guarantee outstanding performance as a transparent electrode while conducting polymers, and the carbon-based materials are in trouble with their insufficient electrical and optical performance^13–16^.

For the application of AgNW electrodes in various types of devices such as thin-film transistors^17,18^, bioelectric devices^19–22^, OLEDs^23,24^, and wearable sensors^25,26^, it is essential to pattern AgNWs into a particular form. Universally adopted methods for patterning AgNWs include microchannel wetting^27^, inkjet printing^28^, photolithography^29^, laser ablation patterning^30^, dip coating^31^, vacuum filtration^32^, and spray coating^33^. However, the majority of conventional methods share a common issue that a large amount of AgNWs was wasted for the patterning process. During the photolithography^34,35^ and laser ablation process^36,37^, unused AgNWs were discarded with an etchant or high-energy laser. Dip coating and spray coating^38–41^ with a stencil mask resulted in the deposition of AgNWs on not only the uncovered substrate but also the stencil mask, which would be removed after the patterning process. As the patterned region by the stencil mask enlarges, more AgNWs were discarded, causing the increased expense of the patterning process. As one approach to minimize the loss of AgNW, a vacuum filtration process was introduced to pattern AgNWs on a masked filter and transfer to the desired substrate using the adhesive stamp^42,43^. However, the vacuum filtration process leaves a residue of AgNWs during filtration through the filter or transfer step on the adhesive stamp. As another approach, there were efforts to recycle the AgNW solution. However, the recycling process could harm the electrical or optical characteristics of AgNWs due to the shortening of AgNW length or surface morphology change^44,45^.

Herein, we demonstrate a highly efficient patterning process of AgNWs based on electrospray deposition with a patterned ground electrolyte solution (EDGE). Through numerical investigation and experimental validation, we confirmed the selective deposition of electrosprayed AgNW droplets on the patterned grounded electrolyte solution due to the concentrated electric field toward the electrolyte solution, thereby achieving a highly efficient patterning of an AgNWs. Furthermore, to verify the versatility of this process in terms of material and shape, we have designed specific patterns on three different substrates; a poly (methyl methacrylate) (PMMA), a poly (dimethylsiloxane) (PDMS), and a glass beaker. As a result, the highly conductive transparent electrode was successfully fabricated on a 2D flat and 3D curved surface of various substrates. To investigate their potential applications in various forms of electrical devices^46,47^, we have developed a self-powered tactile sensor, which is an essential component in human-machine interactions^48–51^, artificial prosthetics^52,53^, healthcare monitoring^54,55^, and environmental observation^56^. Consequently, we suggest both the practical applicability and distinct advantage of this process compared to the conventional methods mentioned formerly with exceptionally enhanced patterning efficiency.

## Experimental sections

### Materials

AgNW solution (0.5 wt%, 25 nm in diameter, 25 µm in length; Flexio, South Korea) with ethanol (99.9%) was purchased. Then, the solution was diluted with ethanol until the concentration of the solution became 0.25 wt%. A PMMA (Choika Co., South Korea) substrate was cut with the dimension of 25 mm × 25 mm × 3 mm. A PDMS substrate was fabricated by mixing PDMS monomers and curing agents (Dow Corning, USA) at a weight ratio (10:1) and subsequently curing at the temperature of 80 °C for 2 h. A polyvinyl chloride (PVC) adhesive dicing tape (DS Semicon, South Korea) was prepared as a masking tape. FEP (Alphaflon Co., South Korea) film was purchased with a thickness of 0.1 mm.

### Electrospray deposition with grounded electrolyte solution (EDGE) on various substrates

Substrates including a PMMA, flat and sandpaper-molded PDMS substrates, and a glass beaker were treated with an oxygen plasma system (CUTE, Femto Science, South Korea). Oxygen plasma treatment was performed with the constant plasma power of 100 W, the flow rate of air with 20 ccm, and the plasma time of 180 s. The PVC adhesive dicing tape was patterned with a comb, alphabet, and university emblem shape as a masking tape by a vinyl cutter plotter (Silhouette Cameo 4, Silhouette America Co., USA). The patterned masking tape was attached to each substrate so that the entire surface of the substrate was separated into two parts; a mask-covered region and -uncovered region, named as a mask region and a target region, respectively. After that, the electrolyte solution was then selectively placed on the target region by the wettability difference between the plasma-treated target region and the mask region. The AgNW solution was then filled into a 10 ml polypropylene syringe (HENKE-JECT, LCK Tech Co., South Korea) and ejected through a 23-gauge needle at a constant flow rate (0.3 ml h^−1^) by a syringe pump (EP100, NanoNC, Korea). A high voltage of 29 kV was applied between the metal needle and the grounded electrolyte solution by a high voltage supplier (HV30, NanoNC, South Korea). The distance between the needle and the grounded electrolyte solution was set to 19 cm. This process was conducted in a PMMA cube under a temperature of 20–25 °C and relative humidity of 40–50%. After that, the patterned mask was carefully removed with tweezers, and the deposited AgNWs were annealed at 100 °C for 1 h to thermally reinforce AgNW junctions to increase of electrical conductivity of an AgNW electrode.

### Numerical simulation

A numerical simulation of the electric field was conducted with COMSOL Multiphysics software (Version 5.0, USA). To investigate the role of the grounded electrolyte solution during the EDGE process, we adopted two different collector systems with and without electrolyte solution on the mask-uncovered region, namely the target region of the PMMA substrate. The electrolyte solution was assumed as 0.01 M KCl solution. Mobile ions in the electrolyte solution can be explained based on the Boltzmann equation, contributing the space charge density ρ(*x*), as follows:

1 $$\uprho \left(\mathrm{x}\right)= -2e{c}_{0}\mathrm{sinh}\left(\frac{e}{{k}_{B}T}\phi \left(x\right)\right)$$

where e is the electron charge, c_0_ is the salt concentration, k_B_ is the Boltzmann’s constant, *T* is the room temperature, and ϕ is the electric potential. A comb-shaped PVC masking tape with a thickness of 80 µm and the relative permittivity (ε_r_) of 4.0 was attached on a non-conductive substrate (PMMA; ε_r_ = 3.0). The width of a comb line was 4 mm, and the comb spacing was 4 mm. The electrospray deposition condition for the numerical simulation was the same as the experiment, including the applied voltage (29 kV) and the distance between the needle tip and the substrate (19 cm).

### Characterization of AgNW electrodes

The AgNW electrode with a comb shape was fabricated on a PMMA substrate with an electrospray deposition time of 30 min by the EDGE process. The width of the comb line and the comb spacing were 500 µm and 1000 µm. Then, the patterned AgNW electrode was observed with a DSLR camera (EOS-M50, CANON, Japan), a microscope (JP/BX43, Olympus, Japan). A scanning electron microscope (SEM; Supra 25, Zeiss, Germany) was used to examine AgNW networks. The coverage ratio of AgNWs on the PMMA substrate was calculated by measuring the covered area by AgNWs based on ImageJ. The optical transmittance was measured using a UV/Visible/NIR spectrophotometer (V-770, Jasco, Japan). The light transmittance was obtained over the wavelength region of 350–700 nm. The sheet resistance was measured based on a 4-wire resistance measurement technique using a digital multimeter (DMM 6500, Keithley, USA). To demonstrate the controllability of the EDGE process, the transmittance and sheet resistance of the AgNW electrode was measured with different electrospray deposition times from 10 to 40 min. The electrical properties of an AgNW electrode deposited on 3 mm thick PDMS substrates by the EDGE process were evaluated under the mechanical tests of cyclic bending test, cyclic stretching test, and tensile test using a universal testing machine (QM-100). During the cyclic bending test, the AgNW electrode was bent from flat to 1/3 cm^−1^ curvature for 1000 cycles. The cyclic stretching test was performed by stretching the AgNW electrode with 5% strain for 1000 cycles. Both cyclic tests were progressed with AgNW electrodes on the PDMS substrate with a dimension of 10 × 2 cm^2^ by 30 mm/s. The tensile test was conducted by stretching the AgNW electrode with a speed of 15 mm/min until fracture occurred. During all three mechanical tests, the relative resistance of the AgNW electrode was measured by utilizing the digital multimeter holding both ends of the sample.

### Fabrication and performance measurement of a self-powered tactile sensor array

A 3 × 3 tactile sensor array including 9 square units with a dimension of 1 × 1 cm whose spacing between units was 5 mm was arranged and connected to the external wires at the edge of the array through thin extension lines with 1 mm width. The tactile sensor array was fabricated with the EDGE process on a 5 × 5 cm FEP film. Copper wires were attached to the end of the extension lines by using an aluminum adhesive tape for electrical connection. Then, patterned AgNW electrodes were encapsulated by a PDMS layer with a thickness of 0.5 mm. The output voltage of the fabricated tactile sensor was measured using an oscilloscope (DSOX1204G, Keysight, USA). As a self-powered tactile sensing system, each unit of the tactile sensor array was connected to a 3 × 3 light-emitting diode (NSPG500DS, Nichia, Japan) array mapping the same location via a copper wire connected to each unit.

## Results and discussion

**Figure 1:**
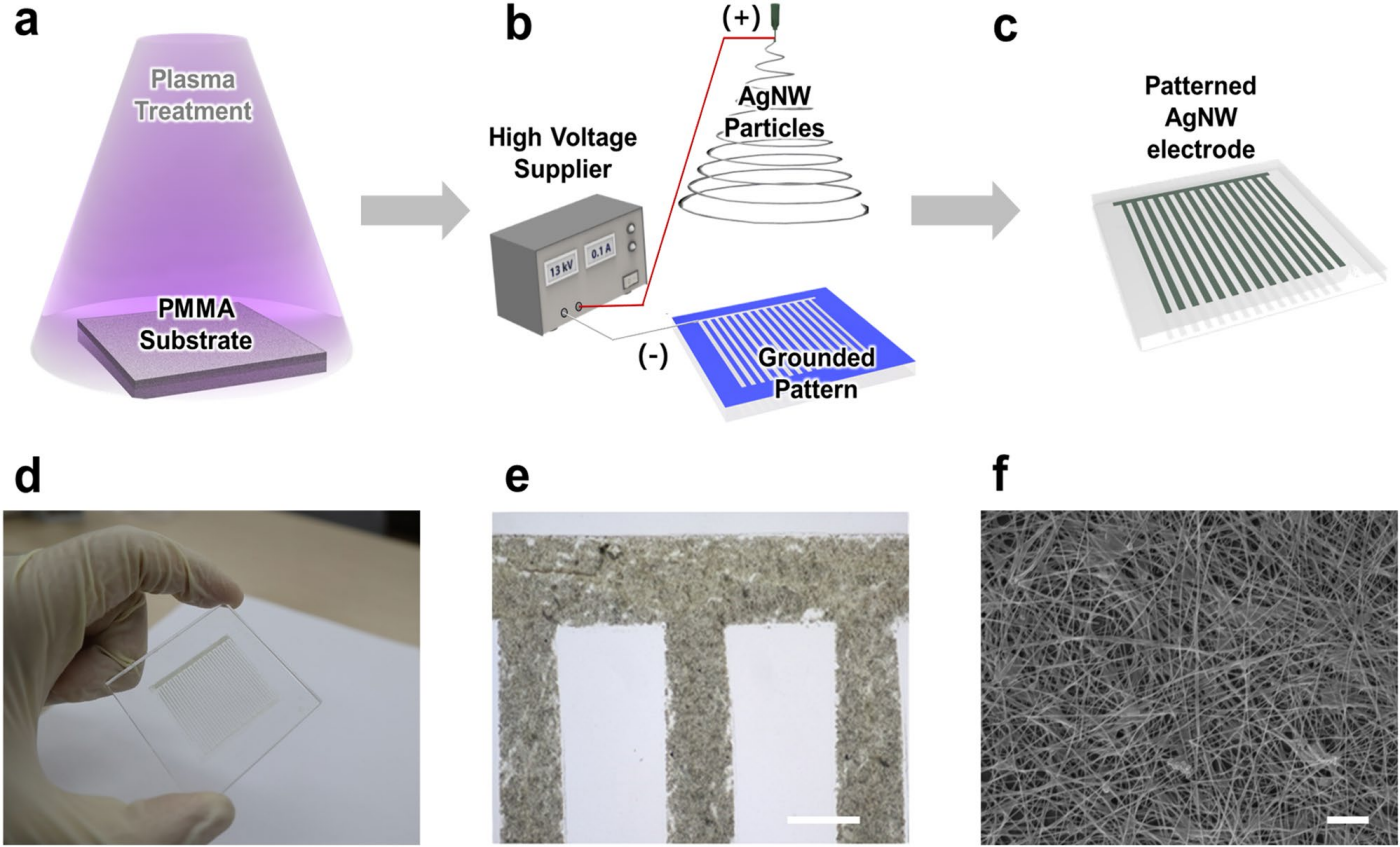
The schematic diagram of the EDGE process and multi-scale observation. (**a**) An oxygen plasma treatment to the PMMA substrate. (**b**) The electrospray deposition process of the AgNW solution with the patterned grounded electrolyte solution. (**c**) The patterned AgNW electrode by the EDGE process. (**d**) The comb-shaped AgNW electrode on a PMMA substrate by the EDGE process. (**e**) The enlarged image of the patterned AgNW electrode with a well-defined edge. (**f**) SEM image of the AgNW electrode. Scale bars are 500 µm (**e**) and 1 µm (**f**).

Figure 1 shows the sequence of the overall EDGE process. The first step of the EDGE process is oxygen plasma treatment to change the wettability of the substrate into hydrophilic (Fig. 1a). The high energy level of the plasma generally leads to the oxidation of the substrate with hydroxyl groups (–OH). For example, the oxygen plasma treatment develops hydroxyl groups at the expense of methyl groups (–CH_3_) in the case of PMMA and PDMS and SiO_2_ in the case of a glass surface, respectively^57,58^. As the hydroxyl groups are polar in nature, which means that the intermolecular bonding between the hydroxyl group and water molecular is stronger than Van der Waals force between water molecules, and thus, the oxygen plasma treatment renders the substrate hydrophilic. Then, a patterned masking tape was attached to a plasma-treated substrate. We used hydrophobic PVC tape as a mask in this study^59^. The major role of the masking tape is selectively positioning the electrolyte solution on the mask-uncovered hydrophilic region of the substrate. Due to the wettability difference between the plasma-treated target region of the substrate and the masking tape, the electrolyte solution was selectively positioned on the target region^60^. Plus, the masking tape prevents the deposition of AgNWs on the undesired region of the substrate. Even if AgNW droplets are attracted to the grounded electrolyte solution as described previously, there still exists a possibility that AgNW droplets could be deposited on the unexpected region. Thereby, the mask forestalls the development of the short circuit leading to the fatal malfunction of electronic devices. The second step is the electrospray deposition process of the AgNW solution (Fig. 1b). In this stage, the metal needle and the patterned electrolyte solution were connected to the anode and ground of the high voltage supplier, respectively. Then, the high voltage was applied to form the Taylor cone at the tip of the needle, and subsequently, highly charged AgNW droplets were ejected. As charge density rises with the evaporation of ethanol in the AgNW droplet, Coulombic repulsion eventually overcomes the liquid’s surface tension, resulting in Coulombic fission of the droplet into micro/nano-sized particles due to Rayleigh instability. Then those particles were deposited on the selectively positioned electrolyte solution, which serves as the temporal ground^61^. Lastly, to increase the electrical conductivity of an AgNW electrode, the deposited AgNWs were then annealed and finally became a patterned AgNW electrode (Fig. 1c).

The comb-shaped AgNW electrode on a PMMA substrate was exhibited in Fig. 1d–f. The whole image of the AgNW electrode and the magnified image of the interconnected region of the comb-shaped AgNW electrode were shown in Fig. 1d,e, respectively. As shown in Fig. 1e, the AgNW electrode could be patterned into a micro-scale with a well-defined edge. Figure 1f shows an SEM image of the AgNW electrode, which exhibited uniformly deposited AgNWs. The EDGE process stably patterned the AgNW electrode with the minimum feature size of several hundreds of micrometers. We expected that the minimum feature size of the EDGE process would be reduced down to several tens of micrometers with the help of additional techniques, as demonstrated in the previous research of the electrolyte solution-based electrospray deposition process^60^. As an example, we fabricated a simple triangular pattern of an AgNW electrode and found that the tip of the triangular pattern could be down to 50 µm, as shown in Fig. S1. In light of the additional experimental data, we expect that the EDGE process will enable stable patterning with several tens of micrometers through merging with a precise mask fabrication technique. While the EDGE process would not provide the highest resolution compared to other techniques, including photolithography^29,62^, filtration patterning^63,64^, and reverse offset printing^65^. Such resolution of the EDGE process would be sufficient to produce various types of electronic devices, including a strain sensor^66,67^, tactile sensor^54,68^, energy harvester^69,70^, and antenna^71,72^. Furthermore, as the EDGE process could produce a transparent electrode with the minimum use of the AgNWs, we believed that the EDGE process is an efficient process to produce transparent optoelectronic devices.

**Figure 2:**
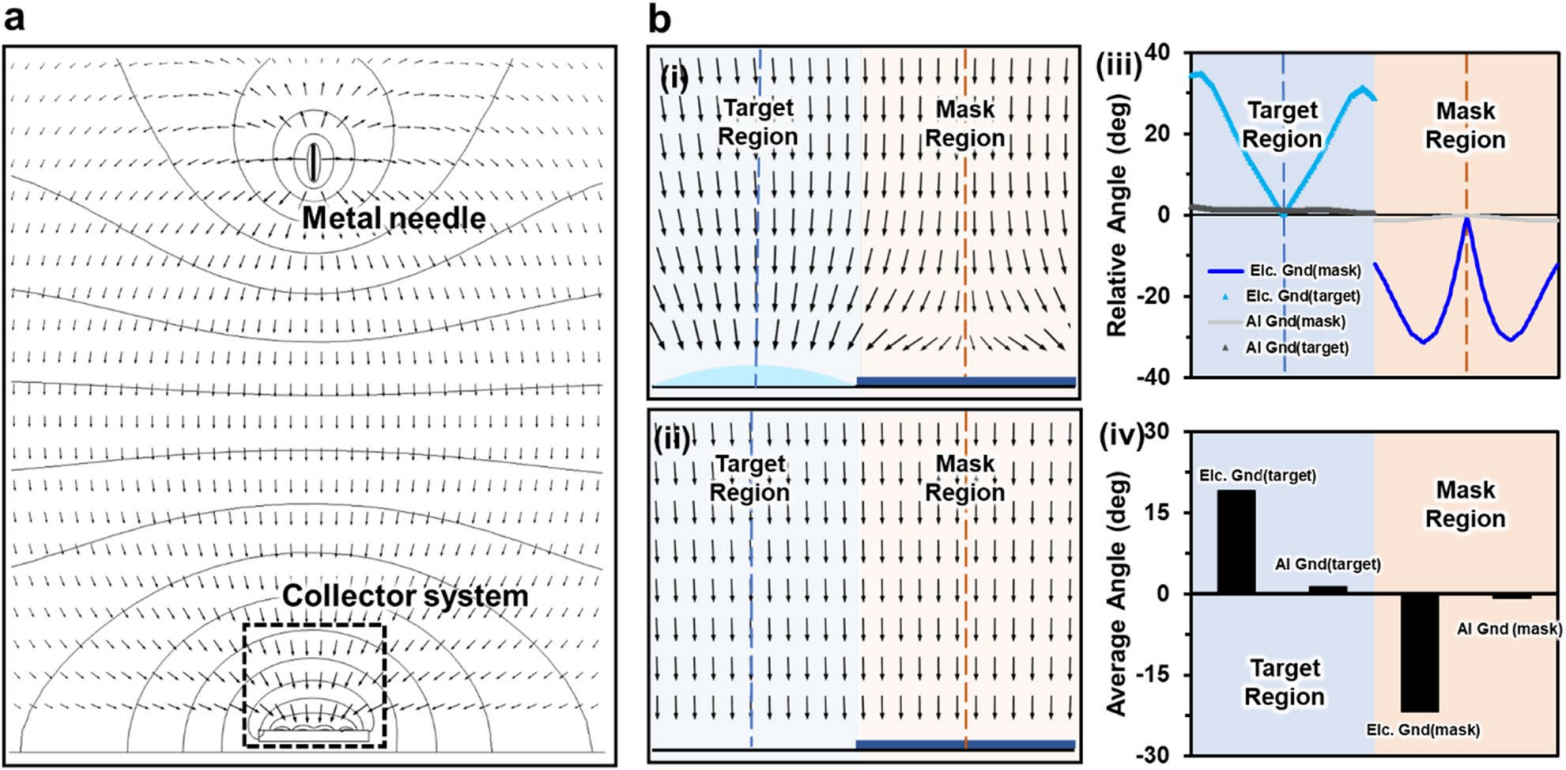
(**a**) Numerical simulation of the electric field in the EDGE process and conventional electrospray process with collector system. A magnified image of the electric field with EDGE collector system (**b**-(i)) and conventional electrospray collector system (**b**-(ii)). The relative angle (**b**-(iii) and the average relative angle of the electric field (**b**-(iv)) to the centerline above a collector system with and without a grounded electrolyte solution.

To verify the highly efficient patterning of the EDGE process, we selected two collector systems with and without electrolyte solution during the electrospray deposition process for the numerical simulation. The tendency of the depositing position of the electrosprayed AgNW droplets was generally determined by the interaction between the electrical charge of the electrosprayed droplets and the electric field. In this sense, the numerical simulation of the electric field has been utilized to support the deposition tendency of electrosprayed AgNW droplets on the collector. Figure 2a shows the layout of the EDGE process with the collector system. The electric fields were concentrated toward the collector system, and thus, the electrosprayed AgNW droplets would be electrostatically attracted toward the collector system. To compare the attraction of the electrospray AgNW droplets toward the collector system with and without electrolyte solution, we evaluated the direction of the electric field toward the target region and the mask region, respectively.

Figure 2b-(i) has shown the direction of the electric field above the target region where the grounded electrolyte solution was placed and the mask region where a masking tape had adhered. The electric field above the target region was concentrated towards the grounded electrolyte solution. On the other hand, the direction of the electric field above the mask region was outward from the center of the mask region. In other words, electrosprayed AgNW droplets would be tended to be deposited more on the target region and less on the mask region. Figure 2b-(ii), representing the conventional collector system without electrolyte solution, shows the direction of the electric field above the target and the mask region. Unlike the collector system with grounded electrolyte solution, the electric field direction showed no significant difference between the target region and the mask region. Thus, electrosprayed AgNW droplets were expected to be analogously deposited both on the target and mask region.

To quantitively evaluate the concentration of electric field, we defined two centerlines located at the center of the target region and the mask region, respectively, as shown in Fig. 2b-(i). And, the value of the relative angle of the electric field was defined as the angle between the centerline and the electric field. Thus, the sign of the relative angle was set to positive or negative when the electric field was closer or further away from the centerline, respectively. To do so, we defined two independent orthogonal coordinate systems that represent the target and mask regions, respectively, where each centerline of the target and mask regions was used as the y-axis (*x* = 0). After that, a relative angle (*θ*_*r*_) is defined as below:

2 $${\theta }_{r} \left(deg\right)=\left(\frac{\pi }{2}-{{\cos}}^{-1}\frac{\overrightarrow{E} \cdot \overrightarrow{\varepsilon }}{\left|\overrightarrow{E}\right|\left|\overrightarrow{\varepsilon }\right|}\right)$$

where $\overrightarrow{\varepsilon }$ is a unit vector of (− 1, 0) when x ≥ 0 and (1, 0) when x < 0, and $\overrightarrow{E}$ is the electric field vector. The positive relative angle implies that the electrosprayed AgNW droplet will be attracted toward the centerline. Conversely, the negative relative angle implies that the electrosprayed AgNW droplet will be repelled from the centerline. Figure 2b-(iii) quantitatively shows the relative angle of the electric field with the collector system with and without grounded electrolyte solution. For the case of the collector system with grounded electrolyte solution, referring to the EDGE process, the relative angle increased as the distance from the centerline increased in the target region. This indicates that even at a position far from the centerline of the target region, electrosprayed AgNW droplets will be deposited on the target region by the electrostatic force directed toward the target region. However, for the case of the collector system without electrolyte solution, the relative angle was maintained at around 0°. The relative angle of near 0° implies that the electrosprayed AgNW droplets will be deposited on the collector system without any preferential attraction to neither the target nor the mask region. Lastly, Fig. 2b-(iv) shows the average relative angle with the collector system with and without electrolyte solution. For the substrate with the grounded electrolyte solution, the average relative angle displayed 19.13° and − 21.67° for the target and mask region, respectively. Contrarily, for the substrate without electrolyte solution, the average relative angles of the target and mask region were 1.30° and − 0.84°. This relative angle difference between the electrolyte solution ground and conventional ground suggests that the major amount of electrosprayed droplets will be deposited on the target region than the mask region by the EDGE process. Due to the low relative angle difference, conventionally electrosprayed particles will be deposited almost equally on both the target region and mask region. In this regard, we could conjecture that the EDGE process would tremendously decrease the unwanted deposition of AgNWs droplets at the mask region, thereby achieving the efficient patterning of an AgNW electrode.

**Figure 3:**
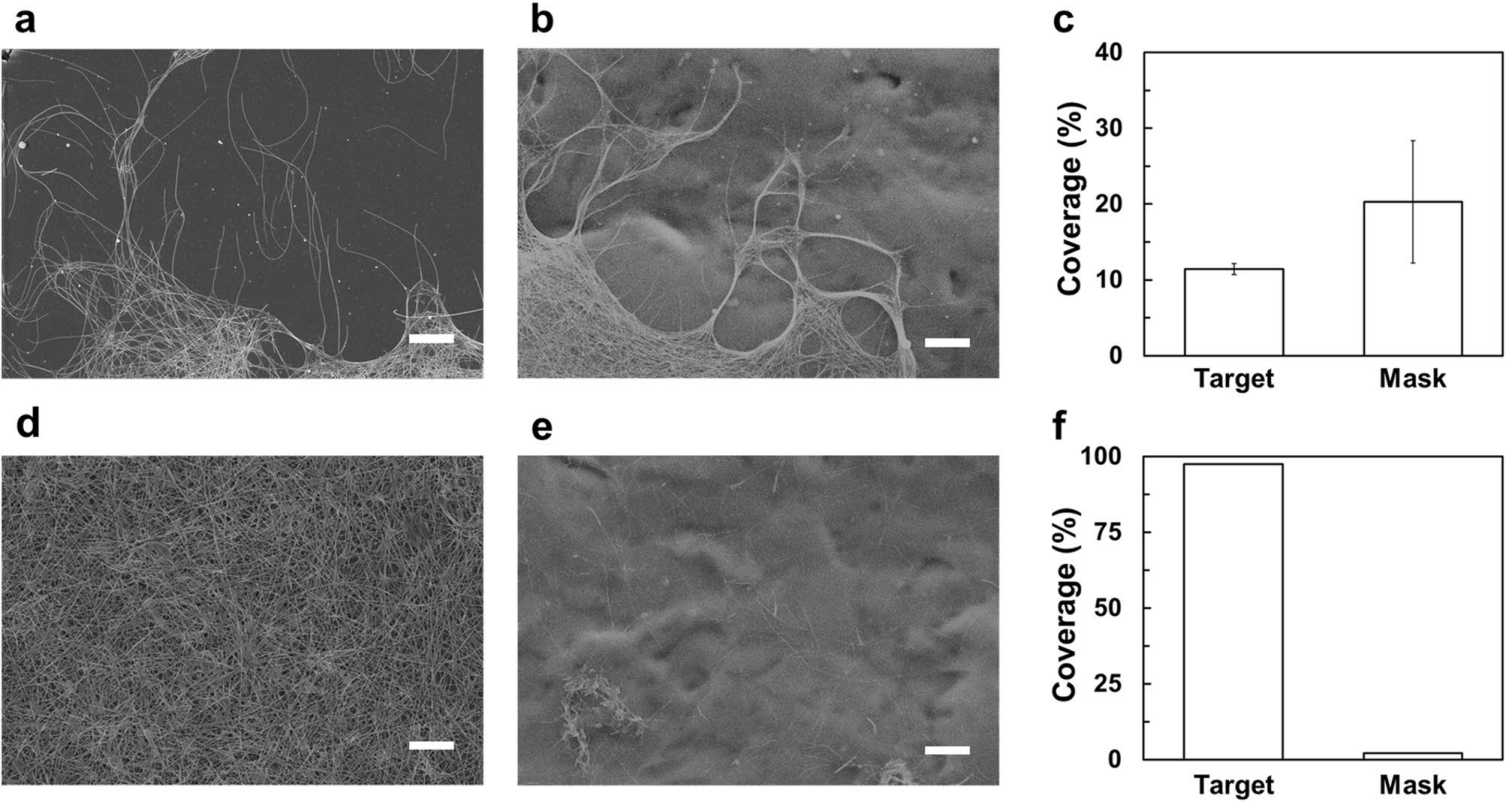
SEM images and the coverage ratio of the AgNWs deposited on the target region and the mask region by the conventional electrospray deposition process (**a**–**c**) and the EDGE process (**d**–**f**), respectively. All scale bars are 5 µm.

We compared the proposed EDGE process with the conventional electrospray deposition. Figure 3 shows SEM images of the AgNWs deposited on the target and mask region by the conventional electrospray deposition and the EDGE process. For the case of conventional electrospray deposition without electrolyte solution, irregular deposition of AgNWs was observed on the target region, which is the main cause of the short of an electric circuit (Fig. 3a). As demonstrated by numerical simulation, AgNWs were also deposited on the undesired mask region by the conventional electrospray deposition (Fig. 3b). We could observe that more AgNWs were deposited on the mask region compared to the target region. The PVC masking tape has a higher dielectric constant compared to that of the PDMS substrate, and the mask region is slightly higher than the target region due to PVC masking tape. Such characteristics made it easier for AgNWs to be deposited on the mask region compared to the target region. Considering that the masking tape would be detached after the fabrication process, it is believed that a significant amount of unused AgNWs would be wasted. The AgNWs were covered only 11.43 ± 0.73% and 20.30 ± 8.07% of the target region and mask region as shown in Fig. 3c, respectively, which implies that many of the AgNWs were not deposited on the substrate due to the electrostatic repulsion between the deposited and coming AgNWs. On the other hand, for the proposed EDGE process, uniform and highly dense networks of AgNWs were observed on the target region leading to the low sheet resistance (Fig. 3d), whereas a sparse amount of AgNWs was deposited on the mask region (Fig. 3e). In Fig. 3f, AgNWs were deposited on the target region almost 100%. Conversely, AgNWs hardly deposited in the mask region. Considering that the target region was fully covered by AgNWs through visual inspection, the coverage ratio of AgNWs on the target region was assumed to be 100%. The density of AgNWs deposited on the mask and target region showed a tremendous difference from Fig. 3c,f, which demonstrated that the EDGE process could dramatically reduce the deposition of AgNWs on the undesired mask region. Furthermore, the EDGE process, which utilized the electrolyte solution collector, greatly increased the coverage ratio at least 6 times higher compared with the conventional electrospray deposition. Thus, we believed that the EDGE process achieved highly efficient patterning of AgNWs.

**Figure 4:**
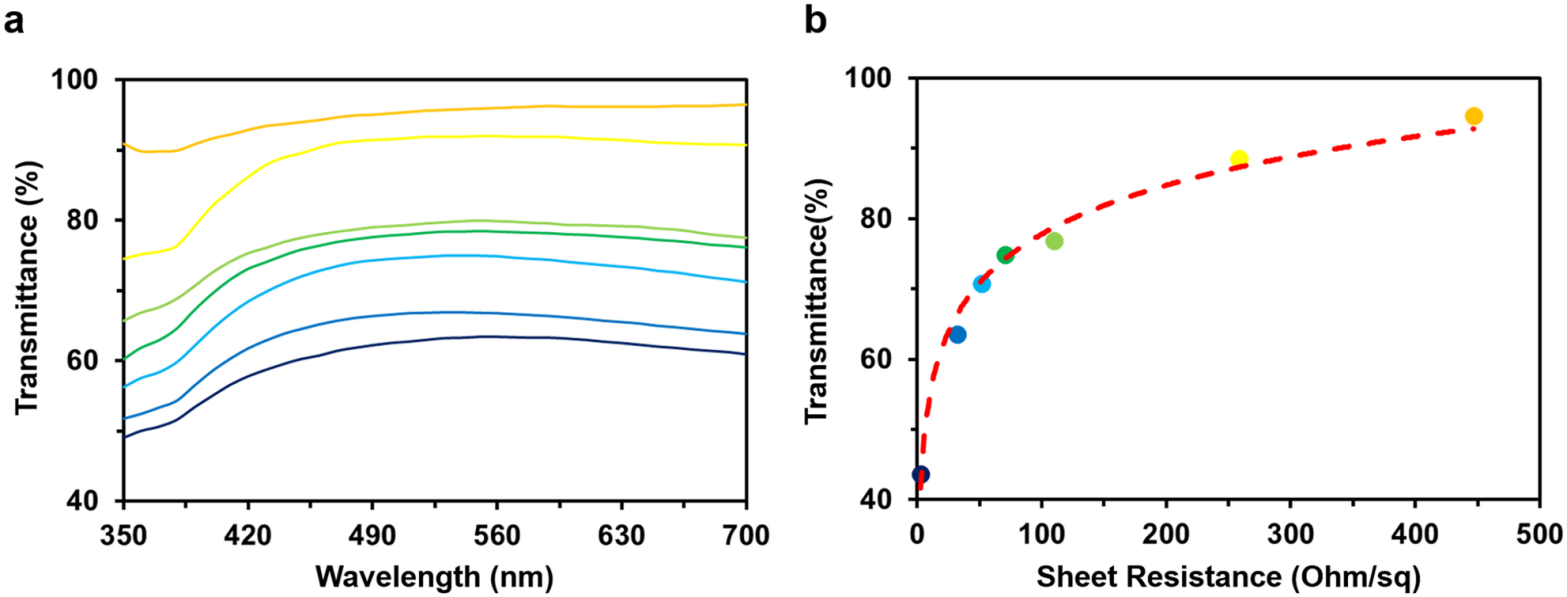
The correlation of (**a**) wavelength and (**b**) sheet resistance with the transmittance of electrosprayed AgNW electrodes by the different electrospray deposition times from 10 to 40 min.

Figure 4a shows the transmittance of electrosprayed AgNW electrodes over 350–700 nm for different electrospray deposition times from 10 to 40 min. The corresponding average light transmittances for each electrospray deposition time were from 94.5 to 43.6%. Figure 4b shows the correlation of the sheet resistance (*R*_*S*_) and average light transmittance of the AgNW electrodes prepared with different electrospray deposition times. The descending sheet resistances were observed from 446.9 Ω/sq with the highest transmittance of 94.5% to 2.7 Ω/sq with 43.6% (Fig. 4b). The light transmittance of the AgNW electrode increases logarithmically with the increase of *R*_*S*_. The higher light transmittance implies that the lower number of AgNWs were deposited on the substrate, and thus, the increase in the light transmittance caused the decrease in the conductivity and the increase in the sheet resistance. By adjusting the electrospray time in the EDGE process, the network configuration of AgNWs could be made dense or thick, and the sheet resistance could also be changed to the desired value. However, light transmittance and electrical conductivity are dependent, so that the electrospray deposition time must be set in consideration of the trade-off characteristic between the two properties.

**Figure 5:**
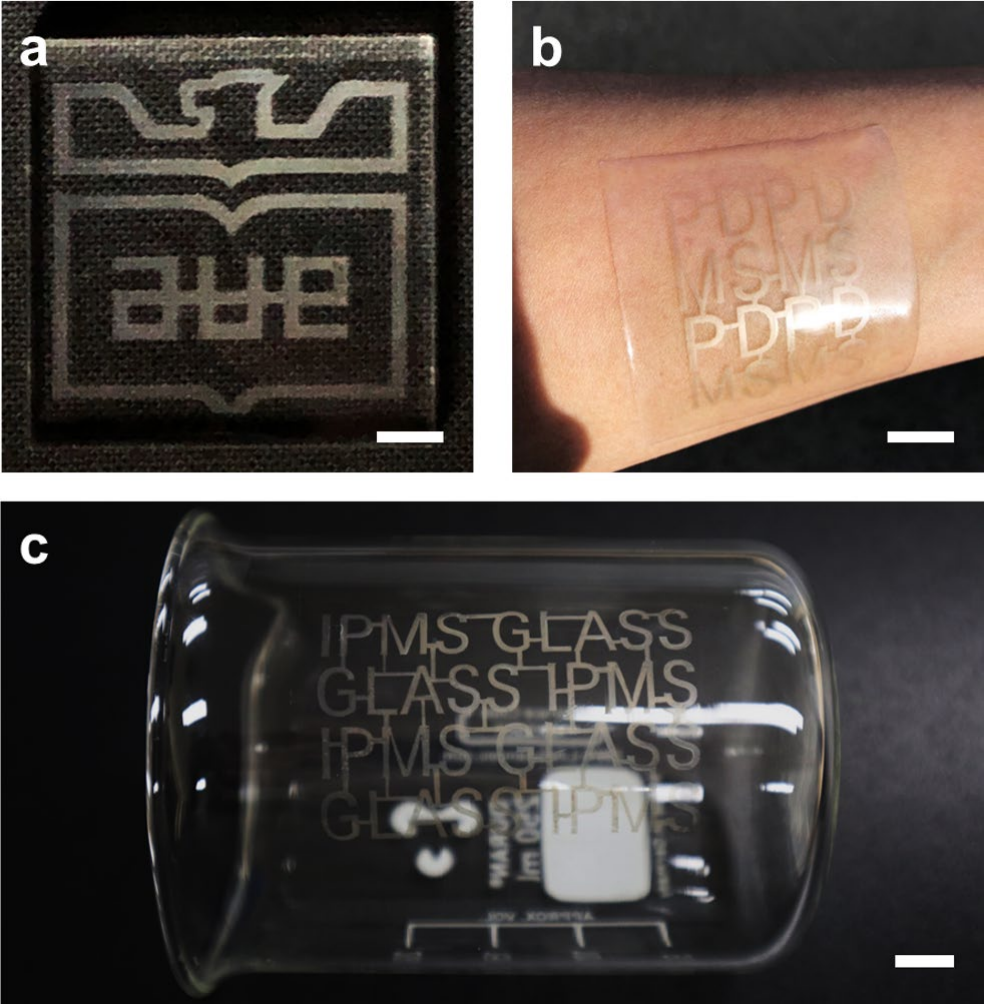
Patterned AgNW electrodes with a university emblem shape on the PMMA substrate (**a**), alphabet-matrix patterns on the PDMS substrate (**b**), and on the curved surface of the glass beaker (**c**). Scale bars are 3 mm (**a**) and 1 cm (**b**,**c**).

For the verification of the pattern controllability in terms of the material of the substrate and the shape of the pattern, AgNWs were deposited on various substrates, as shown in Fig. 5a–c. To demonstrate the high degree of freedom of design, an AgNW pattern that has the shape of the university emblem, as shown in Fig. 5a, was deposited on the flat PMMA plate. The well-defined edge and uniformity of the deposited AgNW electrode suggested the reliability of the EDGE process when applied to optoelectronic devices^73,74^. To verify applicability as wearable and stretchable electronics, a PDMS substrate, a promising candidate with its high stretchability and durability, exhibited an alphabet-matrix pattern, as shown in Fig. 5b. Also, for further investigation of the pattern controllability depending on the surface roughness, PDMS substrates molded with #100, #400 and, #800 grit sandpaper were exploited. The boundary features between the mask and the substrate were shown in Figure S2 by the EDGE process. AgNWs were deposited uniformly on the sandpaper-molded PDMS except for interfacial regions, especially at the groove topography. Furthermore, we could roughly measure the resistance in the order of k $\Omega$ with the 2-wire resistance measurement technique, which demonstrated that the EDGE process could produce an AgNW electrode even on the rough surface. Unfortunately, we could not perform a quantitative evaluation of relative resistance by using the 4-wire resistance measurement technique due to the roughness of the microtopography. As shown in Fig. 5c, we have succeeded in the facile and uniform deposition of AgNWs on a curved surface of a glass beaker, whereas most of the patterning methods such as microchannel wetting, inkjet printing, photolithography, laser ablation patterning, dip coating, vacuum filtration, and spray coating have difficult in patterning an AgNW electrode on curved 3D substrates. For the glass beaker we utilized, the electrolyte solution did not flow and was well located due to the difference in wettability between the plasma-treated target region and the masking tape. Apart from this, the electrolyte solution was sometimes flowing down when the height became larger than the case of the glass beaker. We believed that whether the electrolyte solution is flowing down or not is governed by the balance between the surface tension of the solution and the hydrostatic force caused by the height difference. Therefore, the EDGE process could be utilized to various 3D surfaces by exploiting factors that can control surface tension, such as impurity and temperature, and factors that can control hydrostatic pressure, such as density and local gravity of a solution. Considering the broad compatibility with various 2D and 3D substrates, we believed that the EDGE process would be an efficient and effective way to produce a patterned 3D AgNW electrode for various applications^60,75^.

**Figure 6:**
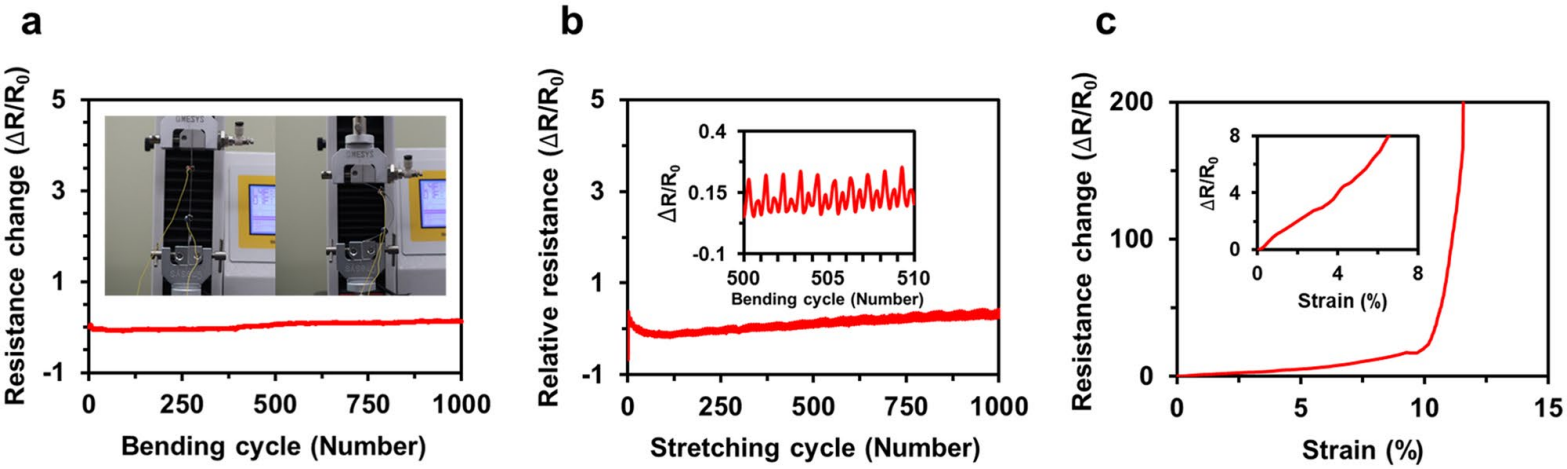
Relative resistance of AgNW electrodes fabricated through the EDGE process. (**a**) the cyclic bending test. Inset: a single cycle image of bending (**b**) the cyclic stretching test with trends of relative resistance. Inset: relative resistance with 10 cycles of stretching test, and (**c**) the tensile test of the AgNW electrode until the break. Inset: linear increase of relative resistance with the strain of 7%.

Figure 6a–c show the change of the resistance under cyclic bending, cyclic stretching, and tensile test of the electrosprayed AgNW electrode, respectively. As a measure for the change of the resistance, we defined the relative resistance of AgNW electrodes expressed as ΔR/R0 = (R − R0)/R0, where R0 is the initial resistance, and R is the measured resistance after the shape change. During 1000 cyclic of the bending tests (Fig. 6a, inset), the relative resistance was slightly altered from − 7.9 to + 14.4%, as shown in Fig. 6a. Furthermore, the relative resistance changes during a single cycle of the bending test maintained a similar trend. From these results, we could conclude that the AgNW electrode maintained its electrical characteristics during the cyclic bending test.

In Fig. 6b, the cyclic stretching test with 5% strain shows that the relative resistance changed from − 0.19 to 0.43 at initial cyclic stretching, we could observe a slight decrease in the relative resistance, but the relative resistance would not be significantly changed throughout the 1000 cycles of the stretching test, and after 500 cycles, the relative resistance seemed to be stabilized to the plateau value. Furthermore, as the cyclic stretching was repeated, the relative resistance change maintained a similar trend as shown in the inset of Fig. 6b. Through the cyclic stretching test, we concluded that the range of applied strain and the stabilization of the relative resistance could affect the fabrication of the desirable strain sensors.

Lastly, the tensile test of the AgNW electrode was conducted to observe the relationship between the strain and electrical resistance in Fig. 6c. The relative resistance value increased linearly until the substrate had a strain of 7% in the inset of Fig. 6c, and then the abrupt increase in the relative resistance was observed after a strain of 12%. The dramatic increase in relative resistance after a strain of 12% of AgNW electrodes is reasonable, given that a similar trend of the electrical resistance could be found in the previous studies^76,77^. Thus, we concluded from three mechanical tests of AgNW electrodes that the AgNW electrodes fabricated by the EDGE process possessed sufficient mechanical stability and durability.

Recently, the tactile sensors which percept various external stimuli are vital components for the next-generation optoelectronic devices. Also, the physical functionalities of the sensors, such as flexibility and transparency, are crucial for the extended design diversity. In this regard, to demonstrate one of the potential applications of the developed AgNW electrodes, the triboelectric-effect-assisted transparent and flexible tactile sensor in a self-powered manner is chosen to be developed. The mechanism of the transparent and flexible self-powered tactile sensor is based on the triboelectric effect^78^. When positively charged material contacts with negatively charged material, triboelectric materials are, the more the triboelectric charges can be generated. The output performance of the self-powered tactile sensor mainly depends on the material selection. Here, the A fluoro (ethylene propylene) (FEP) was selected as a material of the contact layer due to its fluorine (–F) functional group, which can be easily negatively charged with its exclusively high electron affinity. Due to the difference of electron affinity between skin and FEP contact layer, a gentle touch on the FEP contact layer with a finger generates electrical charge pairs. After the separation of the skin and FEP contact layer, the generated charge pairs break, and the net negative electrical charges occur at the FEP contact layer. At this step, due to the presence of the AgNW electrode located beneath the FEP contact layer, positive electrical charges are induced on the AgNW electrode, thereby generating an electrical signal.

**Figure 7:**
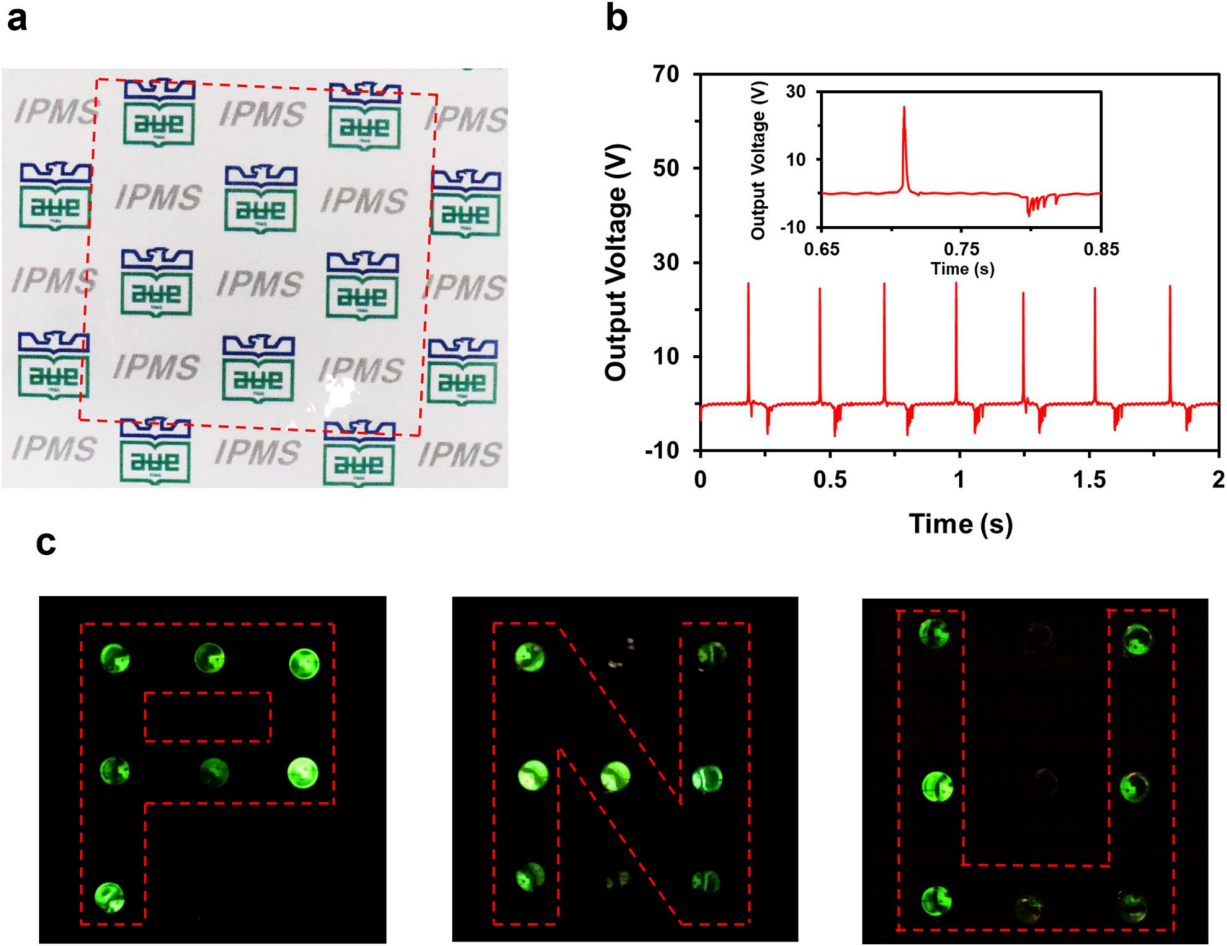
(**a**) A transparent self-powered tactile sensor highlighted by drawing a red-dashed box. (**b**) The electrical responses of the self-powered tactile sensor to touches with a finger. Inset: a single electrical response of the AgNWs electrode. (**c**) The self-powered tactile sensor system powered up a LED array with response to a touch of letter-shaped stamps.

Figure 7a shows the fabricated self-powered tactile sensor array of a PDMS-encapsulated patterned AgNW electrode on an FEP film. Due to the superior light transmittance, it was difficult to distinguish the self-powered tactile sensor from the background, and thus, we highlighted the self-powered tactile sensor by drawing the red-dashed box in Fig. 7a to clarify the edge of the self-power tactile sensor. Not only the self-powered tactile sensor exhibited superior transmittance, but also it generated sufficient electrical signals to detect the touch of the finger. The gentle and continuous touch of the finger on the self-powered tactile sensor produced the output voltage of around 25 V as they contacted and around − 5 V as they separated (Fig. 7b, inset). As with most triboelectric generators, unstable charges were generated during the touching processes, but a constant output voltage could be maintained after repeated touches. To exploit the self-powered characteristic, the self-powered tactile sensing system, which transformed a touch on the self-powered tactile sensor as a LED lightning without any external devices, was developed. When an Al coated stamp with an alphabet shape is contacted with the self-powered tactile sensor, the corresponding LED has lightened (Fig. 7c). Since the stamp was manually transferred and contacted with the FEP contact layer, the non-uniformity and crosstalk of output luminance inevitably occurred. However, the self-powered and position detecting characteristic of the system has a value for further application as a tactile sensor that perceives from simple touch to sliding, multi-touch, hovering, and shear stress by detecting electrostatic field caused by the corresponding stimulations.

## Conclusion

In this study, the EDGE process was introduced as an efficient patterning technique for AgNW electrodes using electrospray deposition. By manipulating the electric field, electrosprayed AgNWs were selectively deposited on various substrates in terms of material, including PMMA, PDMS, and curved glass beaker and shape including 2D flat and 3D curved surface. As a result, the sophisticated and reliable AgNW transparent electrode was fabricated on various types of substrates with the lowest waste of AgNWs. We have fabricated a self-powered tactile sensor array with a simple LED circuit to demonstrate the suitability of the patterned AgNWs. From these results, we believed that the EDGE process could be applicable to various fields, including optoelectric devices, wearable devices, and multifunctional electronic devices.

## Supplementary Information

Supplementary Information.
