# Supplementary material for: Highly efficient patterning technique for silver nanowire electrodes by electrospray deposition and its application to self-powered triboelectric tactile sensor
Source: Sci Rep. 2021 Nov 2;11:21437. doi: 10.1038/s41598-021-01043-6 (PMC8563710; doi:10.1038/s41598-021-01043-6)
Supplement: Supplementary file 1 — Supplementary Information. [file 41598_2021_1043_MOESM1_ESM.pdf]

Supplementary Information for

Highly Efficient Patterning Technique for Silver Nanowire Electrodes by Electrospray Deposition and Its Application to Self-Powered Triboelectric Tactile Sensor

Jin Yeong Song[1,+], Jae Hee Oh[1,+], Dongwhi Choi[2,] Sang Min Park[1,]**

[1]School of Mechanical Engineering, Pusan National University, 63-2 Busan University-ro, Geumjeong-gu, Busan, 46241, South Korea

[2]Department of Mechanical Engineering (Integrated Engineering Program), Kyung Hee University, 1732 Deogyeong-daero, Yongin, Gyeonggi, 17104, South Korea

Corresponding Author

* Prof. Sang Min Park

e-mail: sangmin.park@pusan.ac.kr

** Prof. Dongwhi Choi

e-mail: dongwhi.choi@khu.ac.kr

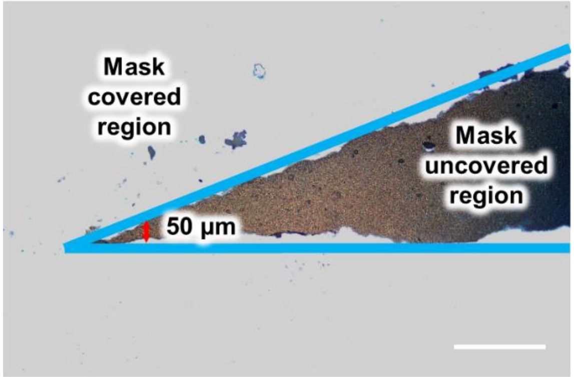

Figure S1. A patterned AgNW electrode with a sharp edge by the EDGE process. The scale bar is 200 μm.

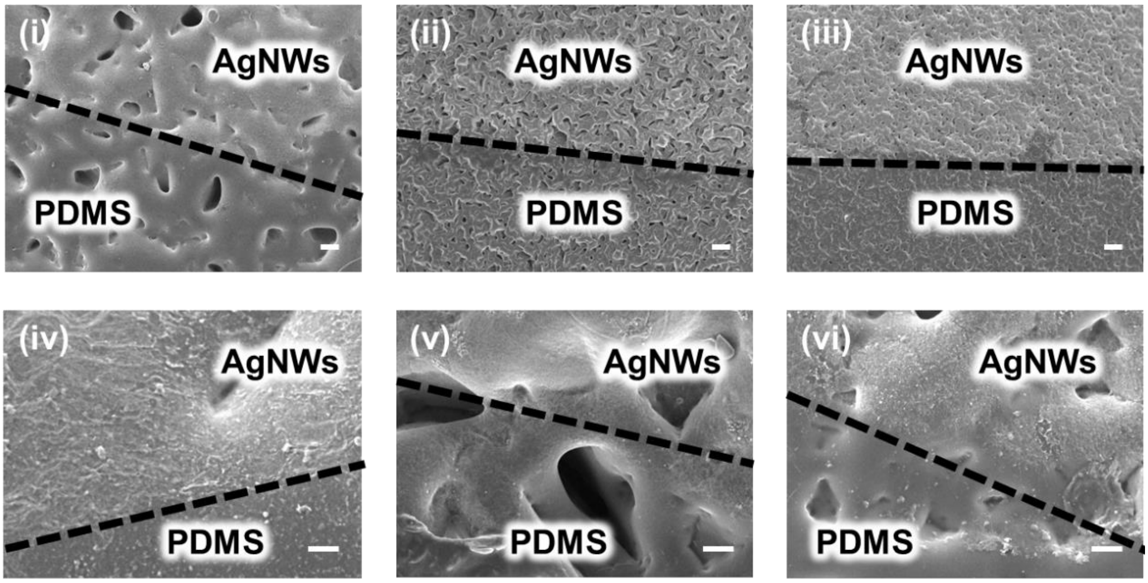

Figure S2. SEM images of electrospray deposited AgNW electrodes on the sandpaper-molded PDMS substrates with #100 grit ((i), (iv)), #400 grit ((ii), (v)), and #800 grit ((iii), (vi)). Scale bars are 100 µm (i, ii, iii) and 10 µm for enlarged SEM images (iv, v, vi).
